# Alcohol consumption promotes colorectal carcinoma metastasis via a CCL5-induced and AMPK-pathway-mediated activation of autophagy

**DOI:** 10.1038/s41598-018-26856-w

**Published:** 2018-06-05

**Authors:** Haodong Zhao, Danlei Chen, Rui Cao, Shiqing Wang, Dandan Yu, Yakun Liu, Yu Jiang, Mei Xu, Jia Luo, Siying Wang

**Affiliations:** 1 Department of Pathophysiology, School of Basic Medicine, Anhui Medical University, Hefei, 230032 China; 2 Department of Pharmacology and Nutritional Sciences, University of Kentucky College of Medicine, Lexington, Kentucky 40536 USA

**Keywords:** Colorectal cancer

## Abstract

There is a definite relationship between alcohol consumption and colorectal cancer (CRC) development. We investigated effect of alcohol consumption on CRC patients’ progression and prognosis by utilizing epidemiological data and found patients with alcohol consumption increased risks of tumor-node-metastasis (TNM), organ metastasis and poorer prognosis. Because their tumor tissues displayed increased expression of C-C chemokine ligand 5 (CCL5), we hypothesized CCL5 might participate in cancer progression in such patients. Ethanol increased the secretion of CCL5 in two CRC cell lines, HT29 and DLD-1. Treatment with CCL5 directly increased migratory ability of these cells, whereas neutralization or knockdown of CCL5 can partially block alcohol-stimulated migration. We further investigated underlying mechanism of CCL5-induced migration. Our results indicated that effects of CCL5 on migration are mediated by the ability of CCL5 to induce autophagy, a cellular process known to be critical for migration. Using high-throughput sequencing and western blotting, we found induction of autophagy by CCL5 takes place via AMPK pathway. Aforementioned ethanol increases CCL5 secretion, CCL5 activates autophagy through AMPK pathway, and autophagy increases migration was confirmed by experiments with autophagy or AMPK inhibitors. To sum up, our study demonstrates that chronic alcohol consumption may promote metastasis of CRC through CCL5-induced autophagy.

## Introduction

Cancer is a major public health problem as it has become the second leading cause of death globally^1,2^. According to a recent report, colorectal cancer (CRC) ranks third among the causes of cancer-related deaths in the USA for the year 2016^3^. Familial CRC accounts for 15–30% of all cases, while lifestyle factors including diet, obesity, smoking, and alcohol consumption may contribute to CRC genesis^4^. Epidemiological investigations indicate that regular alcohol consumption is associated not only with increased risk of developing various cancers, such as lung, gastrointestinal, and breast cancer^5,6^, but also with metastasis^7,8^. Tumor metastasis is a major reason for the failure of cancer treatment and lower postoperative survival rates. Ruiz *et al*.^9^ reported overall five-year survival rates of 69.4% and 67.4% for colon and rectal cancer, respectively. In the presence of concomitant metastasis, the maximum survival rates after surgical intervention do not exceed 20%^10,11^. Therefore, the relationship between alcohol consumption and tumor metastasis should be investigated. In this study, we present the results of a clinical case analysis of the effects of alcohol consumption on CRC metastasis and prognosis.

Chemokines are chemotactic cytokines whose abilities include the promotion of inflammation, immune surveillance, and cancer progression by the induction of chemotaxis^12^. CCL5 (C-C chemokine ligand 5) is a chemokine secreted by T lymphocytes, macrophages, platelets, fibroblasts, epithelial cells, and certain tumors^13^, and has been recently associated in various tumors, including CRC progression and metastasis^14,15^. In the presence of alcohol, tumor cells generate various cytokines and chemokines that accelerate tumor growth^16,17^. Our previous studies also demonstrated that alcohol consumption promoted the progression of tumors^18–20^. These data, combined with the work of Maltby, J. *et al*.^21^ who reported that alcoholic hepatitis patients have higher serum CCL5 levels, led us to the hypothesis that ethanol may enhance CCL5 expression, with the latter possibly mediating, at least partly, the effects of ethanol on tumor progression.

Cell migration is a crucial process for tumor metastasis and a key player in the progress of the disease. Even though there are data linking CCL5 to autophagy in the tumor microenvironment^22^, the exact contribution of CCL5 to autophagy induction has not been investigated yet. In order to investigate this contribution, we applied high-throughput sequencing to screen for possibly related signaling pathways in CRC cells. The role of autophagy in regulating cell migration was further investigated.

## Results

### Alcohol consumption is associated with CRC patients’ progression and poorer prognosis

**Figure 1:**
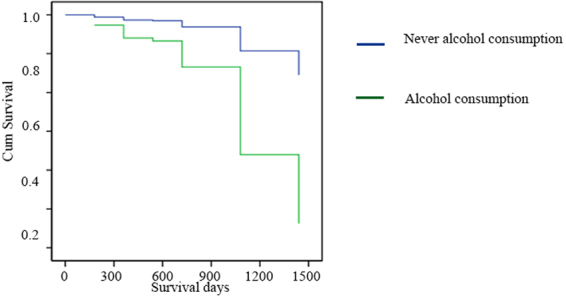
Survival rates of CRC patients and association of alcohol drinking. The overall survival time in 102 CRC patients (43 none-drinkers and 59 alcohol drinkers) in 2012 was analyzed. The post-op survival rate within 5 years was 76.4% for non-drinkers and 36.8% for drinkers respectively. Further multivariate Cox proportional hazaeds model analysis showed of alcohol consumption significantly shortened life span patients (P = 0.001).

To identify a possible correlation between alcohol consumption and prognosis, we retrospectively surveyed 102 CRC patients, among which 69 regularly consumed alcohol for 5–50 years while 43 were nondrinkers (Supplement 1). We performed a multivariate binary logistic regression analysis that was adjusted for sex, age, and IBD, tumor location and tumor grade. Results showed that alcohol-consuming patients had significantly higher possibility of being at an advanced (III-IV) TNM stage compared to nondrinkers (Supplement 2: P = 0.018). Five years after operation, 43 patients had died. The multivariate Cox regression analysis established the TNM stage and alcohol consumption as independent prognostic factors associated with poor survival rates. The hazard ratio of alcohol consumption versus nonalcohol consumption patients was 6.951 (Table 1: 95% CI: 2.509–19.257). Alcohol consumption significantly shortened patients’ life span (P = 0.001, Fig. 1).

**Table 1:** Multivariate survival analysis for survival in 102 CRC patients.

Variables	N	events	HR	95% CI	*P-*Value
**Gender**					
Male	68	14	1		
Female	34	19	1.52	(0.53 4.33)	0.44
**Age**					
≤60	55	12	1		
>60	47	21	1.95	(0.97 3.94)	0.06
**TNM Stage**					
I–II	62	4	1		
III–IV	40	29	8.02	(3.50 18.40)	<0.001*
**IBD**					
negative	85	19	1		
positive	17	14	0.67	(0.27 1.64)	0.38
**Alcohol consumption**					
never	43	4	1		
ever	59	29	6.95	(2.51 19.36)	<0.001*
**Location**					
rectum	52	18	1		
colon	50	15	0.86	(0.38 1.95)	0.73
**Grade**					
highly & moderately	75	16	1		
poorly	27	17	1.04	(0.530 2.21)	1.95

^*^P < 0.001. *Denotes significant difference from control groups.

### Ethanol increases CCL5 expression

**Figure 2:**
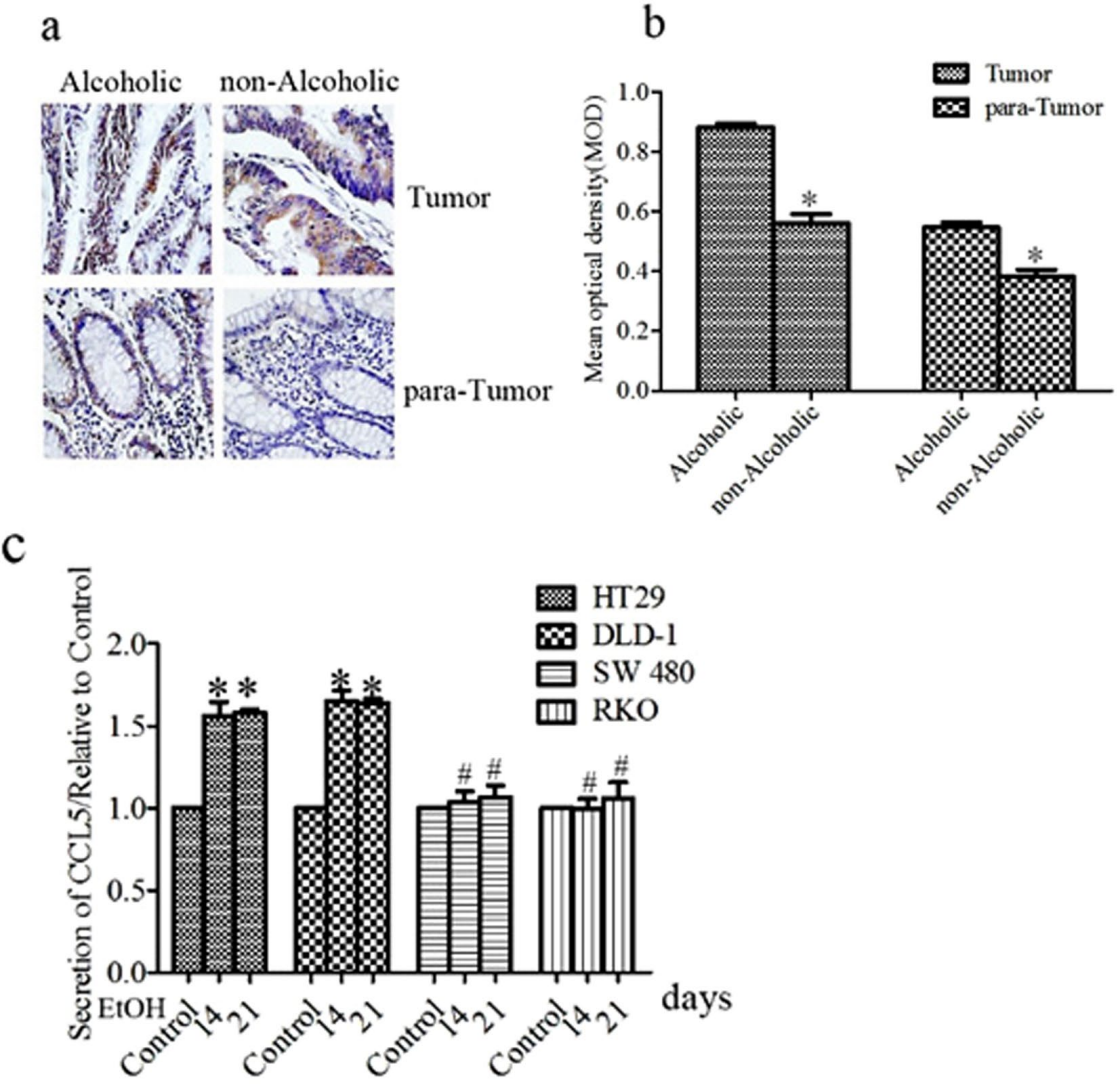
Ethanol increases CCL5 expression. (**a**) Immunohistochemical analysis of CCL5 protein expression in ever alcohol consumption and never alcohol consumption patients tumor tissues (n = 97) (bar: 100 μm). CCL5 indicates brown stain, the former displayed higher levels of CCL5 in both the tumors and the adjacent non-tumor tissues of drinkers. (**b**) Quantitatinve analysis of CCL5 was determined by mean optical density. (**c**) Colorectal cancer cells were exposed to alcohol ((200 mg/ml) for indicated time and conditioned medium were collected and assayed for secreted CCL5 by ELISA. Each data point was the mean ± SEM of three independent experiments and presented relative to the control. *P < 0.05, 1, ^#^P > 0.05.

CCL5 has been implicated in the progression of CRC^23,24^. Since alcohol consumption reportedly promotes the progression of CRC, we examined whether ethanol affects CCL5 expression, using both CRC cell lines and biopsies. A comparative immunohistochemical (IHC) analysis of CCL5 expression in CRC samples between drinkers and nondrinkers revealed that the former displayed higher levels of CCL5 in both the tumors and the adjacent non-tumor tissues. Notably, the high expression of CCL5 in the tumors of drinkers was not restricted to the cancer cells themselves, but also lymphocytes and epithelial cells which can be found inside the tumor (Fig. 2a,b). This suggests that the expression of CCL5 is elevated in the drinking patients’ tumor microenvironment. As the predominant component of tumor tissues is cancer cells, we also examined the effects of alcohol on CRC cell lines. In *in vitro* experiments, a physiologically relevant concentration of alcohol (200 mg/dl) was made the CCL5 expression differently(Supplement 3). We found that the chronic stimulation of two CRC cell lines, HT29 and DLD, with ethanol increased CCL5 secretion (Fig. 2c), confirming that alcohol can increase CCL5 expression in CRC cells. The two cell lines were utilized in the following experiments.

### CCL5 increases the migration of HT29 and DLD-1 cells

**Figure 3:**
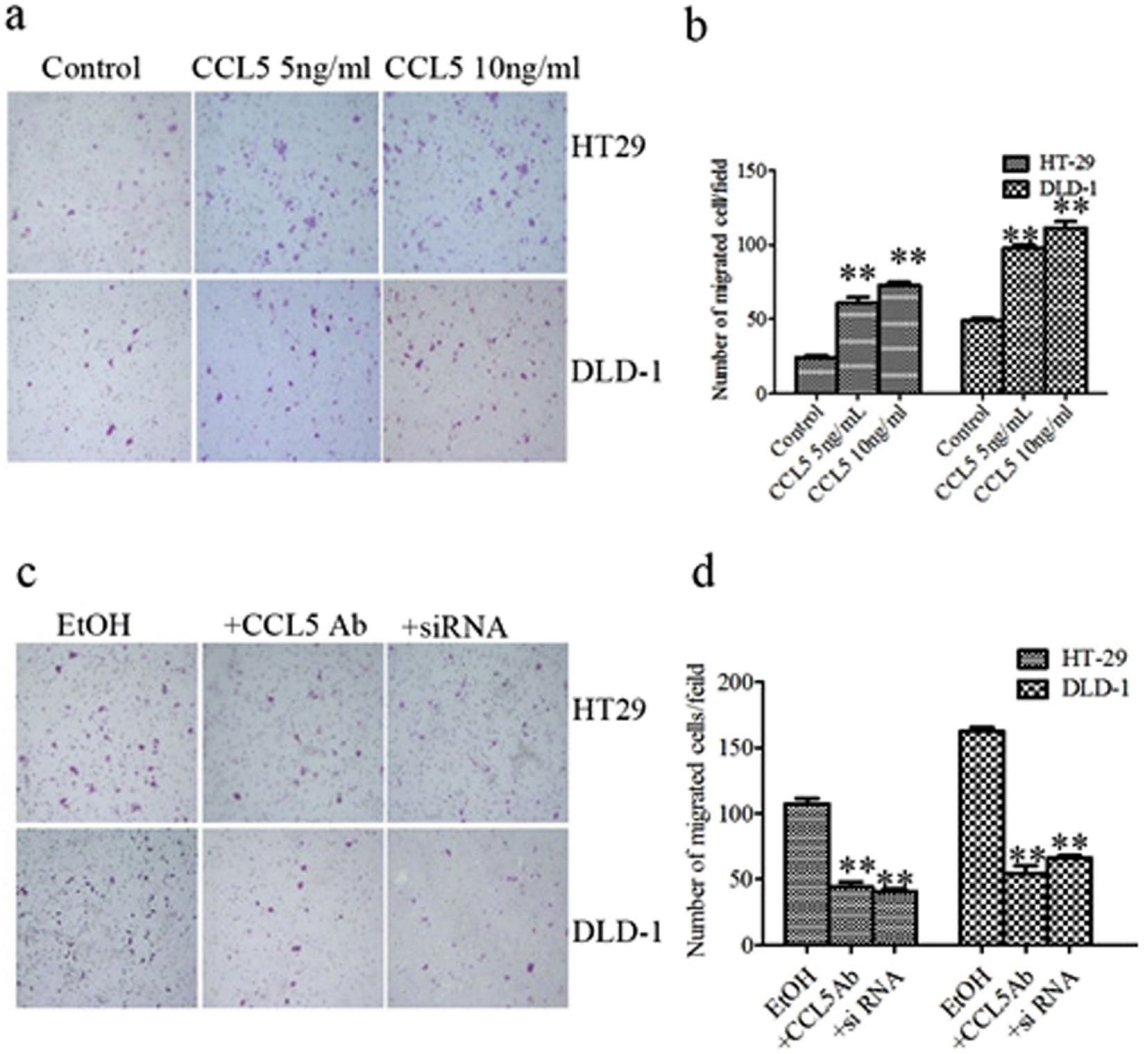
CCL5 increases the migration of HT29 and DLD-1 cells. (**a**) HT29 and DLD-1cells were treated with CCL5 and then assayed for cell migration as described in methods and materials. Representative images of migration are shown. (**b**) Quantification of the migration of HT29 and DLD-1 cells. (**c**) HT29 and DLD-1 cells were exposed to alcohol or pretreated with CCL5 antibody as described in methods and materials. HT-29 and DLD-1 cells were induced by alcohol and transfected with siCCL5. After that, images of cells migrating through the chambers were assayed. (**d**) The cells migrated through the chamber were quantified. Each data point was the mean ± SEM of three independent experiments and presented relative to the controls. **P < 0.01.

Since alcohol increased CCL5 secretion in HT29 and DLD-1 cells, we focused on its role in tumor cell malignant biological behaviors. Proliferation, angiogenesis, and metastasis are all important to cancer progression, and it has been reported CCL5 contributes to these processes^25–27^. In our study, chronic stimulation with CCL5 had no effect on either proliferation of HT29 and DLD-1 cells or angiogenesis by human umbilical vein endothelial cells (HUBVCs) (Supplement 4, 5). In contrast, CCL5 directly increased the migration ability of HT29 and DLD-1 cells (Fig. 3a,b). The inductive effect of CCL5 seemed to reach a plateau at 5 ng/ml CCL5, as increasing the concentration further had little effect on migration. Our experiment proved that ethanol increased cell migration (Fig. 3c,d) and we found that alcohol increased secretion of CCL5 previously. Therefore, we hypothesized that alcohol-increased CCL5 may play an important role in the increase of cell migration ability which caused by ethanol. In order to identify the proportion of the alcohol-induced increased expression of CCL5 on the migration of HT29 and DLD-1 cells, we added CCL5 antibody to the cultures 2 h prior to ethanol exposure that could neutralize the effect of ethanol-increased CCL5, and then observed a decline in the ability of ethanol to enhance cell migration (Fig. 3c,d). At the same time, we knockdown the high expression of CCL5 in HT29 and DLD-1 cells which induced by chronic irritation of alcohol by siRNA (Supplement 6), When the alcohol-increased CCL5 expression was reduced, the cells migration which increase by ethanol were significantly weakened (Fig. 3c,d). Notably, chronic ethanol exposure increased migration to a level higher than the one achieved by direct CCL5 application, because alcohol increased various of cytokines including CCL5 that raised cell migration, but CCL5 neutralization or knockdown abolished the alcohol-induced effect of migration increased partially. These data show that CCL5 may play an important role in alcohol-stimulated migration.

### CCL5 increases migration by inducing autophagy

**Figure 4:**
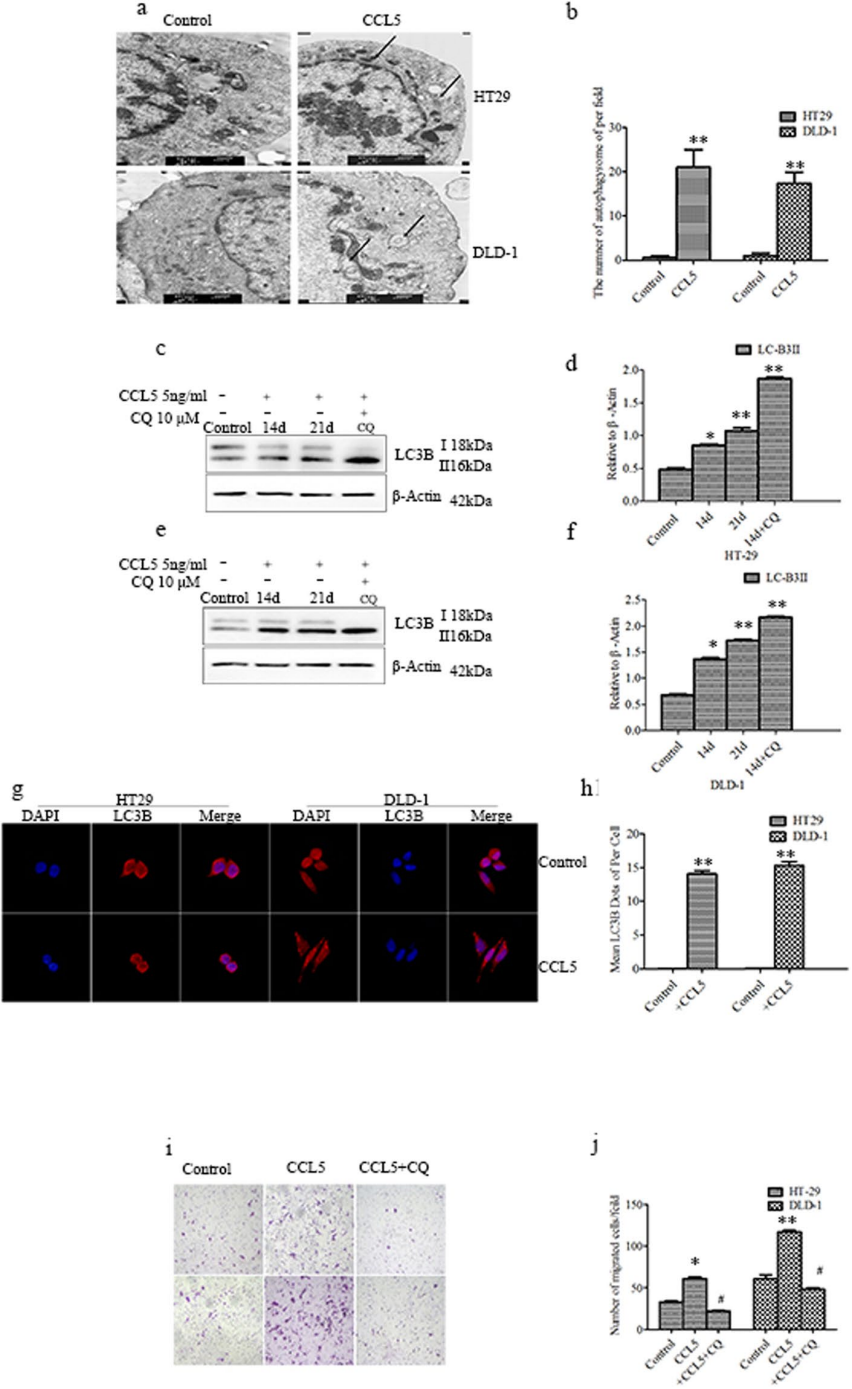
CCL5 increases migration by inducing autophagy. (**a**) TEM images of autophagic vacuoles in HT29 and DLD-1 cells with CCL5 chronic stimulated. Arrows indicate autophagosome, which mainly found in the cells with CCL5 chronic stimulated. (**b**) The number of autophagosome was quantified. (**c**–**f**) HT29 and DLD-1 cells were exposed to CCL5 in presence of CQ or not as described in methods and materials. The expression of LC3B analyzed by western blot. The value of each band indicates the relative expression level after normalizing to the loading control Actin. (**g**,**h**) Cells were double stained by LC3B and DAPI and examined by fluorescence microscopy (400×). Results are the mean ± SEM enumerated from 40 to 60 individual cells. (**i**,**j**) HT29 and DLD-1 cells were exposed to CCL5 and then treated with CQ as described in methods and material. After that, cell migration was assayed. Each data point was the mean ± SEM of three independent experiments and presented relative to the controls. *P < 0.05, **P < 0.01, ^#^P > 0.05.

Autophagy is a cellular catabolic degradation that essential for survival, differentiation, development, and homeostasis^28^. Some studies have reported that autophagy, through which recycling of intracellular metabolites by degrading of damaged organelles and proteins takes place, is required for the motility of tumor cells^29–31^, but the role of CCL5 in autophagy remains poorly understood. Y.F. Peng^32^ reported that inhibited the autophagy in liver cancer cells, can reduce lung metastasis in hepatocellular carcinoma cells in mice. Therefore, we hypothesize that alcohol enhances CCL5 expression and this increase may drives autophagy, which in turn increases the migration. Using transmission electron microscopy (TEM), we observed no autophagosome formation in the control cultures, whereas many autophagosomes were seen in the chronically CCL5-stimulated cultures (Fig. 4a,b). Autophagy is a dynamic and continuous process, so we also examined HT29 and DLD-1 cells at different time points after CCL5 addition and observed, using western blotting, a conversion of LC3B-I to LC3B-II with the passage of time. Moreover, chloroquine (CQ) treatment resulted in a robust autophagic flux, as witnessed by the increase in the autophagosome marker LC3B-II (Fig. 4c–f). Immunofluorescence (IF) microscopy gave consistent results, as visualized LC3B puncta were significantly higher in cells treated with CCL5 (Fig. 4g,h) compared to controls, indicating a transition from LC3-I diffused cytoplasmic staining to LC3-II autophagosome membrane localization, which is characteristic of autophagosome formation. As a whole, these data indicated that CCL5 did induce autophagy in CRC cells. Notably, the inhibition of autophagy by CQ diminished the positive effect of CCL5 on cell migration, indicating that this effect was mediated by the ability of CCL5 to induce autophagy (Fig. 4i,j). Moreover, cells treated with CQ and CCL5 had proliferation rates similar to those of controls (Supplement 7).

### CCL5 induces autophagy through the AMPK signaling pathway

**Figure 5:**
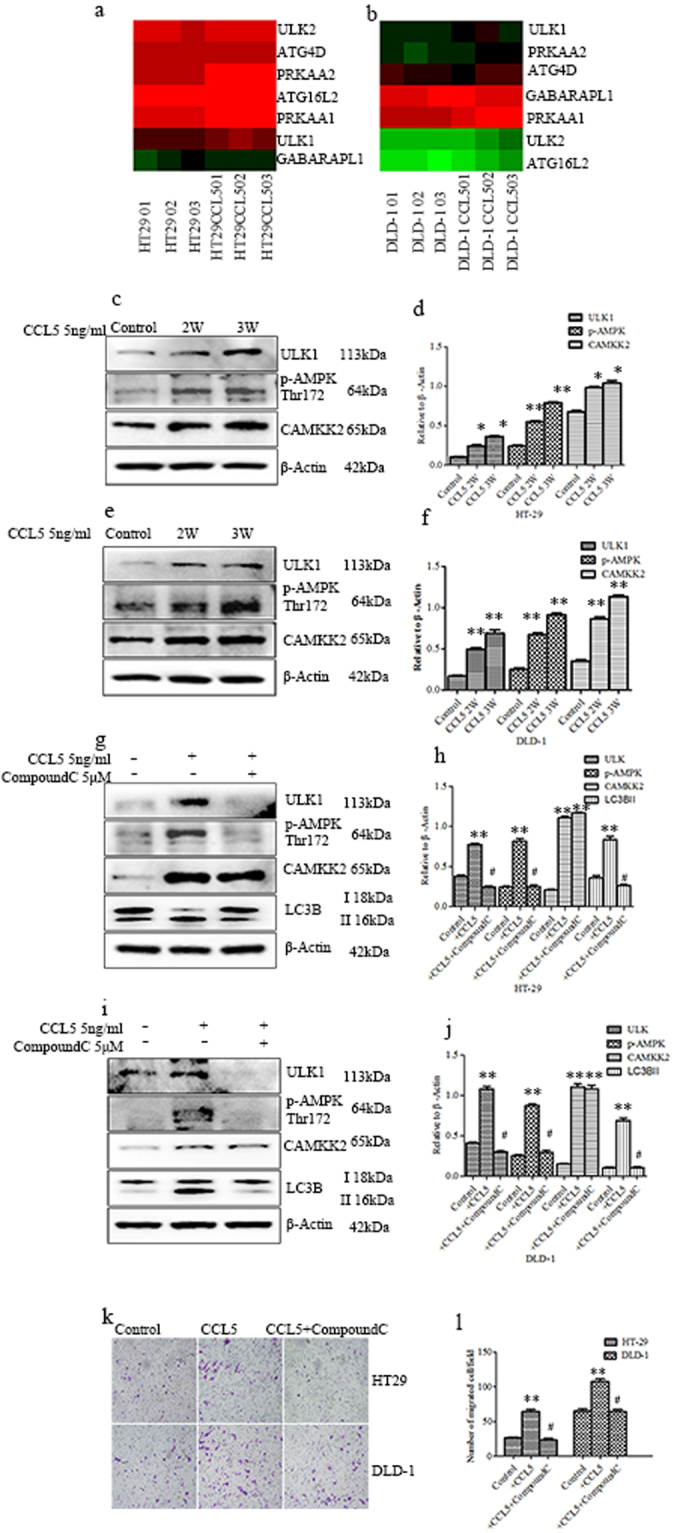
CCL5 induces autophagy through the AMPK signaling pathway. (**a**,**b**) Different expression genes in autophagy path way in HT29 and DLD-1cells with stimulated by CCL5 through High-throughput sequencing. (**c**–**f**) HT29 and DLD-1cells with stimulated by CCL5 as described in methods and materials and then the expression of ULK1, CAMKK2 and phosphorylated AMPK tyrosine 172 were analyzed by western blot. The expression of β-Actin used as a loading control. (**g**–**j**) HT29 and DLD-1 cells were pretreated with Compound C or not and then exposed to CCL5 as described in methods and materials, the expression of ULK1, CAMKK2, LC3B and phosphorylated AMPK tyrosine 172 were analyzed by western blot. The value of each band indicates the relative expression level after normalizing to the loading control Actin. (**k**,**l**) HT29 and DLD-1 cells exposed to CCL5 and then treated with Compound C as described in methods and materials. After that, cell migration was assayed. Each data point was the mean ± SEM of three independent experiments and presented relative to the controls. **P < 0.01, ^#^P > 0.05.

After demonstrating that CCL5 induced autophagy, we investigated the underlying molecular mechanism(s). RNA sequencing is a powerful tool for analyzing of transcriptome and genome in detail. It has revolutionized our current understanding of complex regulation and change with the transcriptome or genome among various treatment conditions^33^. In the present study, we compared the RNA-Seq profiles of CCL5-stimulated and control CRC cells (both HT29 and DLD-1) to discover changes in gene expression, with a focus on genes that have been associated with autophagy. The identified genes are shown in Fig. 4a,b. Among the encoded proteins, PRKAA1 and PRKAA2 are protein kinases that comprise the catalytic alpha subunit of the AMP-activated kinase (AMPK), whose activity is absolutely dependent on the phosphorylation of the α-subunit on Thr172^34,35^, which in turn is mediated by the calcium/calmodulin-dependent protein kinase 2 (CAMKK2)^36^. The ULK family of proteins constitute the initial proteins of autophagy. ULK1 is the predominant isoform involved in autophagy. AMPK regulates autophagy through coordinated phosphorylation of ULK1^37^. Although the exact AMPK-mediated ULK1 phosphorylation site(s) remains unclear, many studies reported ULK1 to increase when autophagy is induced^38,39^. We also observed changes in the expression of genes encoding proteins downstream of ULK1 in autophagy, such as ATG4 and GRBARAPL1 (Fig. 5a,b), but these were not further investigated. The aforementioned changes in the mRNA levels of *PRKAA1*, *PRKAA2*, and *ULK1*, combined with previous reports that CCL5 activates CAMKK2^40,41^, led us to the hypothesis that CCL5 induces autophagy via the AMPK signaling pathway. To test this hypothesis, we first performed western blotting for determining the changes in the protein levels of CAMKK2, p-AMPK α (Thr172), and ULK1, and found all three to be upregulated by CCL5 stimulation (Fig. 5c–f). Moreover, pretreatment of the cells with compound C, a substance commonly used to inhibit the activity of AMPK, attenuated the CCL5-induced increase in the level of p-AMPK α (Thr172) and reverted the conversion of LC3B-I to LC3B-II, indicating that the inhibition of the AMPK also blocked autophagy. As expected, the levels of ULK1, which is located downstream of p-AMPK α (Thr172), were also significantly reduced, whereas the CCL5-induced increase in CAMKK2 levels, which is upstream of the point of inhibition, was not alleviated (Fig. 5g–j). As a whole, these data indicate that CCL5 induces autophagy via the AMPK signaling pathway. Notably, the inhibition of autophagy by compound C also alleviated the CCL5-induced increase in HT29 and DLD-1 cell migration (Fig. 5k,l), whereas changes in cell proliferation were inconspicuous (Supplement 7). Together, these results demonstrate that AMPK signaling plays a crucial role in the migration of CRC cells, at least under the condition of *in vitro* CCL5-induced autophagy.

## Discussion

Many studies show that regular alcohol consumption not only increases the risk of gastrointestinal tumors including colorectal carcinoma but also promotes tumor progression^42,43^. There was wide variation in terms of our study design, the age and gender of study participants, the location and grade of lesion, the units of alcohol measurement and the covariates adjusted for in multivariate models. Nevertheless, we observed that alcohol-consuming CRC patients tended to be at advanced TNM stages and suffered a poorer prognosis compared to nondrinkers, which suggests that alcohol consumption may also promote the progression of existent CRC besides increasing the risk of carcinogenesis. The studies provide evidence for a positive linear relation between alcohol consumption and colorectal cancer. Our clinical data provided a firm foundation for our further experiments and supported our conclusions despite some flaws of this survey. For example, the cohort size was relatively small because it was difficult to recruit female alcohol-drinking CRC patients, as alcohol consumption is much less common among women than men in our country. We identified some of patients clinical trials, but the proportion of such trials still represent a small minority of studies performed within the cancer clinical trials enterprise. In order to reduce the different types of wine and dosage, the sample size of this study is small, so this study is limited to the consumption of white wine and can only be divided into used and never used. In the IHC experiment of pathological specimens, only CCL5 was selected as the target of detection, but ignored some differentially expressed genes between tumors from drinkers vs. non-drinkers which may contribute mediation effects alongside CCL5.Moreover, important risk factors such as smoking, dietary habits, level of education and socioeconomic status were not taken into account in the current study.

The high expression of CCL5 in CRC patients’ tumor tissues with alcohol consumption, combined with the fact that alcohol accelerated CCL5 secretion from HT29 and DLD-1 cells, led us to the hypothesis that CCL5 may contribute to the progression of CRC. Further experiments showed that CCL5 had no effect on HT29 and DLD-1 cell proliferation and HUVEC angiogenesis, indicating that it does not mediate the effects of alcohol on either proliferation or angiogenesis. Alcohol has no significant effect on the proliferation of colorectal cancer cells consistent with what we expected (Supplement 8). In contrast, the migration of HT29 and DLD-1 cells was upregulated by chronic stimulation with CCL5. This result, combined with the observation that CCL5 neutralization with an anti-CCL5 antibody or CCL5 knockdown using siRNA effectively inhibited alcohol-induced cell migration, strongly suggested that CCL5 is a crucial factor in the promotion of CRC by alcohol that acts by increasing cell migration. We have found there were slightly different biological characteristics of four colorectal cancer cells. HT29 cells has the high radiation resistance. DLD-1 cells are derived from patients whose tumors classified is Dukes’ type C. RKO is a low-differentiated colon cancer cell line that contains wild type P53 but lacks the human thyroid receptor nuclear receptor (h-TR beta 1). SW480 is a primary colorectal cancer cell that mutates in the 12th crypto of the Ras gene. The differences between cell lines may be important for the different secretion of CCL5 after alcohol stimulation and the specific mechanism still needs further research.

Ridley, A. J. *et al*.^44^ reported CCL5 increased the migration by binding G-protein-coupled which leading to polarization of the actin cytoskeleton. As increased migration of tumor cells is essential for tumor metastasis, it contributes to cancer development and a poor prognosis^45^ and autophagy is known to play a role in this process as it allows for solid tumor metastasis by recycling metabolites^46^. Thus, we proceeded to investigate the mechanism through which CCL5 directly increased HT29 and DLD-1 cell migration. We demonstrated that CCL5 enhances migration of HT29 and DLD-1 cells and shed additional light on the relationship between CCL5 and autophagy. Our results from TEM, western blotting, and IF indicate that chronic stimulation with CCL5 is sufficient to induce an autophagy flux in HT29 and DLD-1cells.

The regulation of autophagy is a complex process as numerous pathways participate. To clarify the relationship between CCL5 and autophagy, we explored the potential molecular pathway(s) involved in the regulation of autophagy by CCL5 through high-throughput sequencing technology. We analyzed the transcriptomes of control and CCL5-treated HT29 and DLD-1 cells and then screened for differences in the expression of genes associated with autophagy. PRKAA1 and PPKAA2 both belong to the catalytic subunit of AMPK whose enzymatic activity is absolutely dependent on the phosphorylation of Thr172, so we presumed that CCL5 induced autophagy through the AMPK pathway. In agreement with our expectation, the expression of the related proteins increased during the induction of autophagy by CCL5. Additionally, abolishing the activation of p-AMPK using Compound C inhibited the CCL5-induced changes downstream the point of inhibition, whereas upstream elements were not affected. Blocking the AMPK pathway with Compound C also reverted the conversion of LC3BI to LC3BII. Together, these data establish a function for CCL5 as an inducer of autophagy in HT29 and DLD-1 cells via the AMPK signaling pathway. To explore the role of autophagy in metastasis, we blocked autophagy by CQ or the AMKP signaling pathway by Compound C and found that migration was reduced in HT29 and DLD-1 CCL5-stimulated cells, indicating that autophagy may play a crucial role in CRC cell migration.

Pritchard, M.T and Roberson, R.^16,17^ reported that alcohol stimulated cancer cells to secrete variety of cytokines and chemokines, including some inflammatory cytokines and CCL5. It will be more convincing if we get a comparative assessment on other chemokines. The correlation between other chemokines expression and the alcohol consumption of patients will be determined in subsequent investigations. There are crossovers between the receptors and ligands of chemokines, which make the study of chemokine more complicated. While the effects of CCL5 on malignancy may be strongly implicated in multiple myeloma and breast cancer, in which CCL5 promotes both proliferation and migration^47,48^, their contribution in HT29 and DLD-1 cells seems to mainly affect their motility. This suggests that CCL5 regulates the biological behavior of tumor cells through different mechanisms in different cell types.

In summary, our findings illuminate for the first time that alcohol enhances CCL5 expression and this increase drives autophagy, which in turn increases the migration of CRC cells via the activation of AMPK signaling. The targeted manipulation of CCL5-induced autophagy and the AMPK signaling pathway that mediates this induction may be a means of preventing CRC cell migration, which would improve CRC treatment and prevention.

## Material and Methods

### Materials

The MTT assay kit was purchased from Nanjing KeyGEN Biotech (Nanjing, China). Transwells were purchased from BD Biosciences (Bedford, MA, USA). Ethanol, CQ, recombinant human CCL5, and anti-CCL5 antibody were purchased from Sigma-drich (St. Louis, MI, USA). The CCL5 Human ELISA Kit was purchased from CUSABIO (Hubei, China) and *CCL5* siRNA from Santa Cruz Biotechnology (Dallas, TX, USA). Anti-LC3B, were purchased from Cell Signaling Technology (Beverly, MA, USA). Anti-ULK1, anti-phospho-AMPK(Thr172), and anti-CAMKK2 were purchased from Sangon Biotech (Shanghai, China). All other chemicals were obtained from Sigma-Aldrich unless stated otherwise.

### Clinical patient data

The medical records of 102 CRC patients that had undergone anatomical tumor resection were obtained from the First Affiliated Hospital of Anhui Medical University between January and December 2012. Data on major clinical characteristics including alcohol consumption, pathologic stages, and tumor grade were collected. We identified the ever drinkers patients which means consumed alcohol more than 50 g/day for 5–50 years. Survival data were acquired by telephone interviews for all but five patients with whom contact was lost. The pathological sections of the 102 CRC patients were acquired from the pathology department of the First Affiliated Hospital of Anhui Medical University. All methods in the study were carried out in accordance with the latest version of the Declaration of Helsinki, and all experimental protocols were approved with the First Hospital of Anhui Medical University ethics board. Informed consent of the participants was obtained either.

### Cell Culture and Treatments

Four human CRC cell lines (HT29, DLD-1, RKO, and SW480) were cultured in DMEM containing 10% fetal bovine serum and 1% antibiotic/antimycotic solution (Kang Yuan Biology, WH, China) at 37 °C with 5% CO_2_. For alcohol exposure, a sealed container was utilized to maintain a stable concentration of alcohol in the culture medium. A physiologically relevant concentration of alcohol (200 mg/dl) was used in most experiments^49^. For CCL5 neutralization, anti-CCL5 antibody (1 μg/ml) was added to the culture medium 2 h prior to ethanol treatment. For inhibition of autophagy, CQ was added to the culture medium at a concentration of 10 μM, 7 h before exposure to CCL5. For blocking AMPK signaling, compound C was added to the culture medium at a concentration of 5 μM, 2 h prior to CCL5 treatment.

### Quantification of *CCL5* by RT-PCR

After ethanol treatment, total RNA was extracted using the TRIzol reagent (Invitrogen, Carlsbad, CA, USA) according to the manufacturer’s instructions. Next, cDNA was synthesized from 1 microgram RNA using the Reverse Transcription System according to the manufacturer’s instructions. The relative expression levels of mRNA were normalized to the housekeeping gene *GAPDH* and compared with those of the control group. Amplification was performed using the Dream Tag Green PCR Master Mix (Thermo Fisher Scientific, Waltham, MA, USA) according to the manufacturer’s instructions. The following forward (F) and reverse (R) primers were used: *GAPDH*-F, 5′-CCTTCATTGACCTCAACTAC-3′; *GAPDH*-R, 5′-CTCCTGGAAGATGGTGATGG-3; *CCL5*-F: 5′-CGCTGTCATCCTCATTGCTA-3′; *CCL5*-R, 5′-CCATTTCTTCTCTGGGTTGG-3′; Each experiment was replicated at least three times.

### Cell Migration

HT29 and DLD-1cells were plated at a density of 1 × 10^6^ and 2 × 10^5^ cells/well, respectively; in the upper chambers of tranwells (pore size, 8.0) in serum free medium. Culture medium containing 10% FBS served as a chemoattractant and was added to the lower chambers. The chambers were maintained at 37 °C in 5% CO2 for 24 h. Cells migrated to the bottom of the filters and were fixed and stained with 0.5% crystal violet. Migrated cells were photographed and counted. Each assay was repeated at least three times.

### Cell Fractionation and Immunoblotting

Western blotting and SDS-PAGE were preformed according to standard procedures. Cells were lysed in RIPA buffer with a protease inhibitor cocktail. Protein samples (10 μg) were subjected to electrophoresis on 8–12% SDS-polyacrylamide gradient gels and then transferred to PVDF membranes. Membranes were incubated with LC3B(1:1000), CAMKK2(1:1000), ULK1(1:1000), p-AMPK(1:500) or β-Actin(1:2000) antibodies overnight at 4 °C. Anti-rabbit or anti-mouse HPR conjugated antibodies were used as second antibodies followed by enhanced chemiluminescence reagent. Protein bands were visualized with the ECL detection system. The blots were quantified with software of ImageJ analyzer software (National Institutes of Health, USA). Each experiment was replicated at least three times.

### Immunohistochemistry

Pathological sections were deparaffinized, antigen-blocked with 10% normal goat serum, and then incubated with primary antibody (anti-CCL5, 1:500) overnight. The next day, sections were incubated with horseradish peroxidase-conjugated goat anti-rabbit IgG for 1 h followed by addition of the avidin-biotin complex. The activation of the enzyme was visualized by 3.3′-diaminobenzidine (DAB). The mean optical density (MOD) of the stained sections was measured using the Image Pro Plus 6.0 software (Media Cybernetics, Rockville, MD, USA).

### Immunofluorescence

LC3B was utilized to evaluate the induction of autophagy in cells stimulated by CCL5. Briefly, cells were fixed in 4% paraformaldehyde, permeabilized with Triton X-100, sealed for 1 hour at room temperature with 10% normal goat serum for blocking non-specific antigen, incubated with primary antibody LC3B (1:200) overnight in a humidified chamber, and then incubated with the FITC-conjugated secondary antibody. The numbers of LC3 puncta were counted under a fluorescence microscope in randomly selected high-power fields.

### Transmission electron microscope

Cells were harvested, fixed in 2.5% glutaraldehyde for 12 h, and post-fixed in 1% osmium tetroxide. Subsequently, cells were dehydrated using ethanol solutions of increasing concentration (50, 70, 90, and 95%) for 15 min each time, then pure ethanol for 45 min, followed by acetone for 30 min. Finally, the samples were embedded in EPOM812. Ultrathin sections (70 nm) were obtained and stained with 2% uranyl acetate for 20 min followed by 1% lead citrate for 5 min. The sections were then observed under a JEM-1230 transmission electron microscope (NESSIAN, Yokohama, Japan).

### High-throughput sequencing and bioinformatics analysis

Total RNA was extracted from CCL5-treated cultures and controls using the TRIzol reagent (Invitrogen, Carlsbad, CA, USA) and sent to Guangzhou RiboBio Co., Ltd (Guangzhou, China) for high-throughput sequencing using the Illumina HiSeq 2500 platform. Total RNA was isolated from cells/tissues using the Trizol (invitrogen) according to the manufacturer’s protocol. RNA purity was assessed using the ND-1000 Nanodrop. Each RNA sample had an A260:A280 ratio above 1.8 and A260:A230 ratio above 2.0. RNA integrity was evaluated using the Agilent 2200 Tape Station (Agilent Technologies, USA) and each sample had the RINe above 7.0. Briefly, mRNAs were isolated from Total RNA using NEBNext® Poly(A) mRNA Magnetic Isolation Module (illumina, USA) and fragmented to approximately 200 bp. Subsequently, the purified RNAs were subjected to first strand and second strand cDNA synthesis following by adaptor ligation andenrichment with a low-cycleaccording to instructions of NEBNext® Ultra™ Directional RNA Library Prep Kit for Illumina(NEB, USA). The purified library products were evaluated using the Agilent 2200 Tape Station and Qubit®2.0(Life Technologies, USA) and then diluted to 10 pM for cluster generation *in situ* on the HiSeq2500 single-end flow cell followed by sequencing (1 × 50 bp) on HiSeq2500. RNA-Seq data were aligned to the PPI dataset of the KEGG database as previously described^50^. Other bioinformatics analyses were performed using the GLBbase toolkit^51^.

### Statistical analysis

Statistical analysis was performed by SPSS 16.0 (IBM Corporation, Armonk, NY, USA). The correlations between tumor characteristics and alcohol consumption were analyzed by Chi-square test, whereas multivariable Cox proportional hazards regression was used for the correlation between survival prediction and alcohol consumption. A value of P lower than 0.05 was considered statistically significant.

## Electronic supplementary material

Supplement figures and tables
