# Author Correction: Alcohol consumption promotes colorectal carcinoma metastasis via a CCL5-induced and AMPK-pathway-mediated activation of autophagy

**DOI:** 10.1038/s41598-025-00082-7

**Published:** 2025-05-23

**Authors:** Haodong Zhao, Danlei Chen, Rui Cao, Shiqing Wang, Dandan Yu, Yakun Liu, Yu Jiang, Mei Xu, Jia Luo, Siying Wang

**Affiliations:** 1 Department of Pathophysiology, School of Basic Medicine, Anhui Medical University, Hefei, 230032 China; 2 Department of Pharmacology and Nutritional Sciences, University of Kentucky College of Medicine, Lexington, KY 40536 USA

Correction to: *Scientific Reports* 10.1038/s41598-018-26856-w Published Online 05 June 2018.

This Article contains errors.

In Figure 3a, the panels for HT29 CCL5 10 ng/mL and DLD-1 CCL5 10 ng/mL are inadvertently duplicated from the HT29 CCL5 5 ng/mL and DLD-1 CCL5 5 ng/mL conditions, respectively. The correct Figure 3 and its accompanying legend appear below.

**Fig. 3:**
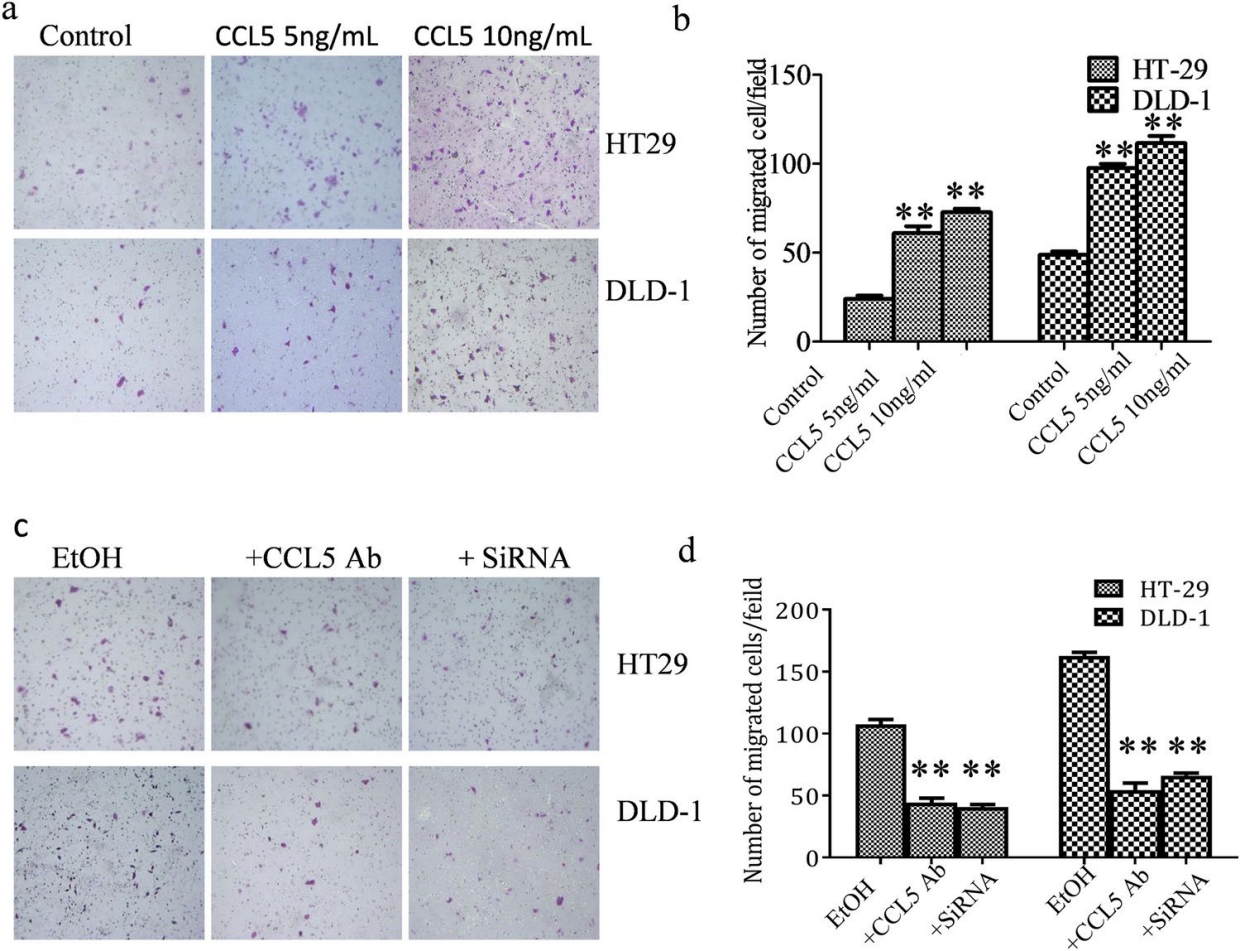
CCL5 increases the migration of HT29 and DLD-1 cells. (**a**) HT29 and DLD-1cells were treated with CCL5 and then assayed for cell migration as described in methods and materials. Representative images of migration are shown. (**b**) Quantification of the migration of HT29 and DLD-1 cells. (**c**) HT29 and DLD-1 cells were exposed to alcohol or pretreated with CCL5 antibody as described in methods and materials. HT-29 and DLD-1 cells were induced by alcohol and transfected with siCCL5. After that, images of cells migrating through the chambers were assayed. (**d**) The cells migrated through the chamber were quantified. Each data point was the mean ± SEM of three independent experiments and presented relative to the controls. **P < 0.01.

In Figure 5k, the panel for DLD-1 CCL5 + CompoundC is inadvertently duplicated from the DLD-1 control group. The correct Figure 5 and its accompanying legend appear below.

**Fig. 5:**
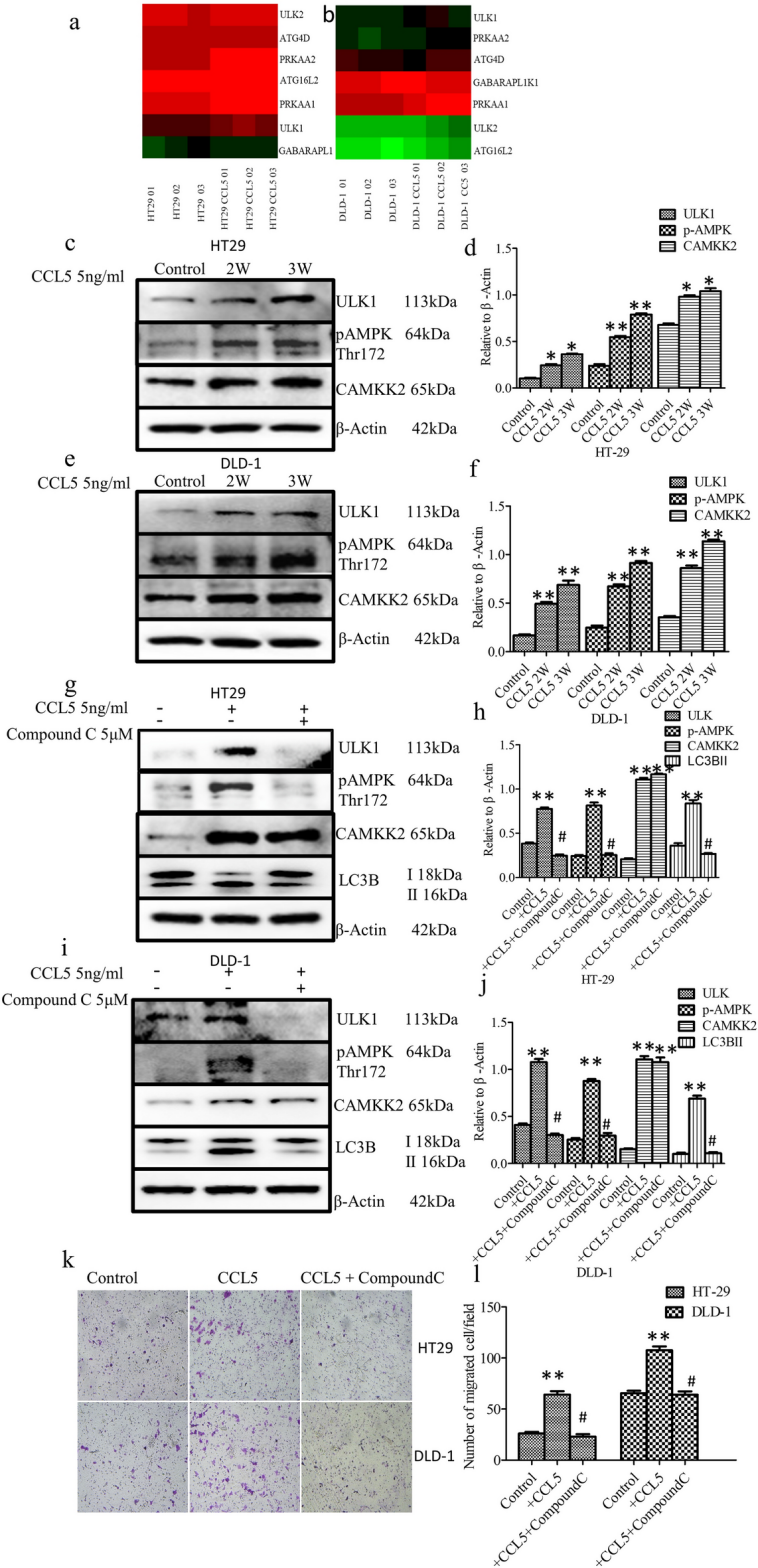
CCL5 induces autophagy through the AMPK signaling pathway. (**a**,**b**) Different expression genes in autophagy path way in HT29 and DLD-1cells with stimulated by CCL5 through High-throughput sequencing. (**c**–**f**) HT29 and DLD-1cells with stimulated by CCL5 as described in methods and materials and then the expression of ULK1, CAMKK2 and phosphorylated AMPK tyrosine 172 were analyzed by western blot. The expression of β-Actin used as a loading control. (**g**–**j**) HT29 and DLD-1 cells were pretreated with Compound C or not and then exposed to CCL5 as described in methods and materials, the expression of ULK1, CAMKK2, LC3B and phosphorylated AMPK tyrosine 172 were analyzed by western blot. The value of each band indicates the relative expression level after normalizing to the loading control Actin. (**k**,**l**) HT29 and DLD-1 cells exposed to CCL5 and then treated with Compound C as described in methods and materials. After that, cell migration was assayed. Each data point was the mean ± SEM of three independent experiments and presented relative to the controls. **P < 0.01, ^#^P > 0.05.

These changes do not affect the conclusions of the Article.

