# Supplementary material for: Alcohol consumption promotes colorectal carcinoma metastasis via a CCL5-induced and AMPK-pathway-mediated activation of autophagy
Source: Sci Rep. 2018 Jun 5;8:8640. doi: 10.1038/s41598-018-26856-w (PMC5988731; doi:10.1038/s41598-018-26856-w)
Supplement: Supplementary file 1 — Supplement figures and tables [file 41598_2018_26856_MOESM1_ESM.pdf]

Alcohol consumption promotes colorectal carcinoma metastasis via a CCL5-induced and AMPK-pathway-mediated activation of autophagy

Haodong Zhao[1], Danlei Chen[1], Rui Cao[1], Shiqing Wang[1], Dandan Yu[1], Yakun Liu[1], Yu Jiang[1], Mei Xu[2], Jia Luo[2]* and Siying Wang[1]*

Supplement 1 Major clinical characteristics of 102 CRC patients

Covariates		All case (n=102)
Sex	Male	68(66.7)
	Female	34(33.3)
Age(yr)	Mean±SD	57.44±12.848
TNM stage	I	7(6.9)
	II	55(53.9)
	III	30(29.4)
	IV	10(9.8)
Metastasis	Negative	63(53.9)
	Positive	39(38.2)
Grade	High &Moderately	75(73.5)
	Poorly	27(26.5)
IBD	Negative	70(68.6)
	Positive	32(31.4)
Location	Colon	50(49.0)
	Rectum	52(51.0)
Alcohol consumption	Never	43(42.2)
	Ever	59(57.8)

Supplement 2 Univariate analysis of tumor characteristics and alcohol consumption in the 102 CRC patients

Variables		Alcohol Consumption n (%) Never	Ever	X^2	*P* value
Gender	Male	17(25)	51(75)	24.62	0.000**
	Female	26(76.5)	8(23.5)		
Age	≤60	30(54.5)	25(45.5)	7.513	0.060
	>60	13(27.2)	34(72.3)		
IBD	Negative	34(40.0)	51(60.0)	0.973	0.324
	Positive	9(52.9)	8(47.1)		
TNM Stage	I	6(85.7)	1(14.3)	10.04	0.018*
	II	26(47.3)	29(52.7)		
	III	8(26.7)	22(73.3)		
	IV	3(30.0)	7(70.0)		
Location	Colon	21(42.0)	29(58.0)	0.001	0.975
	Rectum	22(42.3)	30(57.7)		
Grade	High& Moderately	37(49.3)	38(50.7)	5.984	0.014*
	Poorly	6(22.2)	21(77.8)		
Metastasis	Negative	32(50.8)	31(49.2)	5.040	0.025*
	Positive	11(28.2)	28(71.8)		

*P<0.05

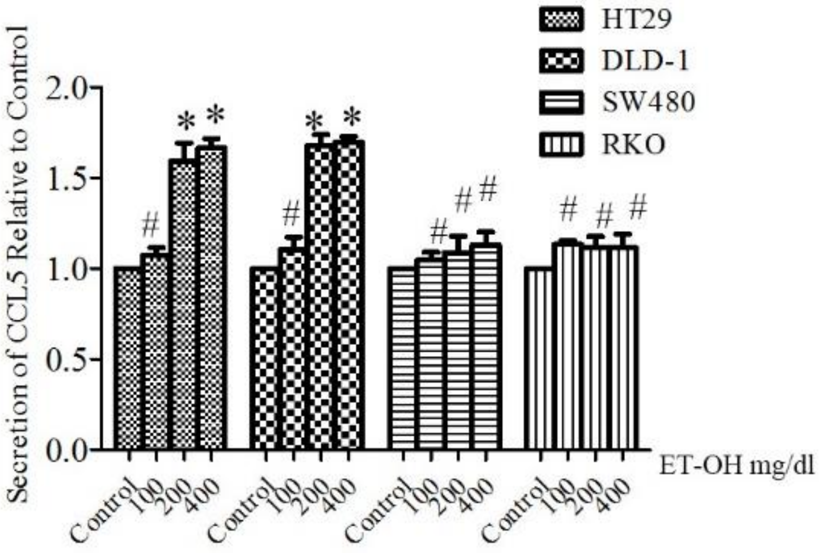

Supplement 3 Colorectal cancer cells were exposed to alcohol with different concentration for 14 days and conditioned medium were collected and assayed for secreted CCL5 by ELISA. Each data point was the mean±SEM of three independent experiments and presented relative to the control.

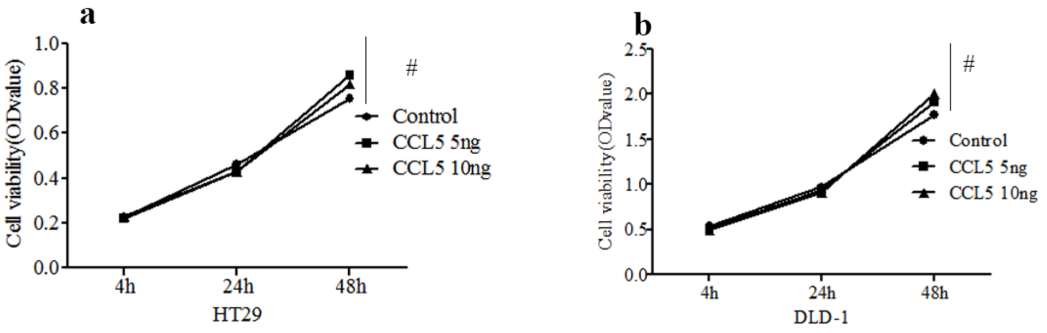

Supplement 4 CCL5 has no effect on CRC cells proliferation（#P>0.05）. Each data point was the mean±SEM of three independent experiments and presented relative to the control value.（#P>0.05 n=3）.

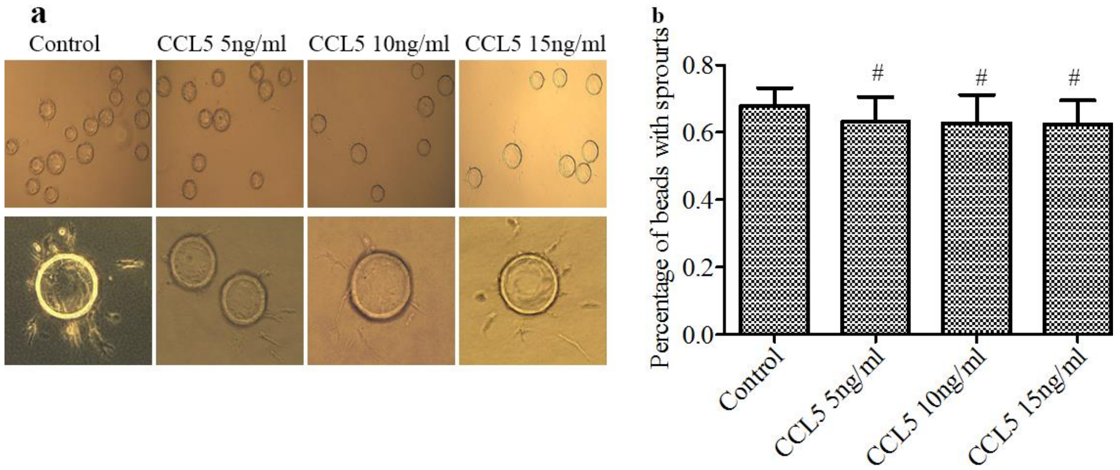

Supplement 5 CCL5 has no effect on angiogenesis（#P>0.05. Each data point was the mean±SEM of three independent experiments and presented relative to the control values.（#P>0.05 n=3）

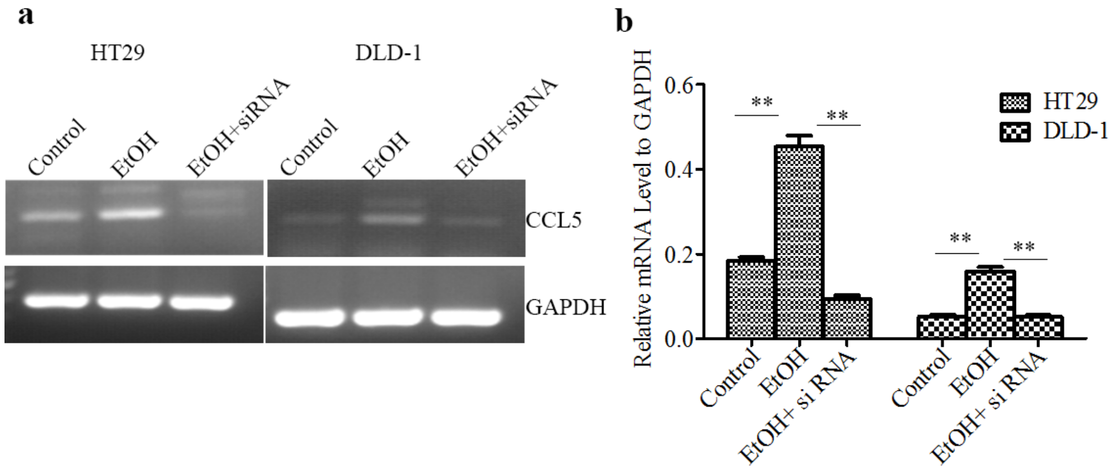

Supplement 6 EtOH stimulated HT29 and DLD-1 cells for two weeks and then transfected with si-CCL5. CCL5 expression was analyzed by RT-PCR. Each data point was the mean±SEM of three independent experiments and presented relative to the control values (**P<0.01 n=3).

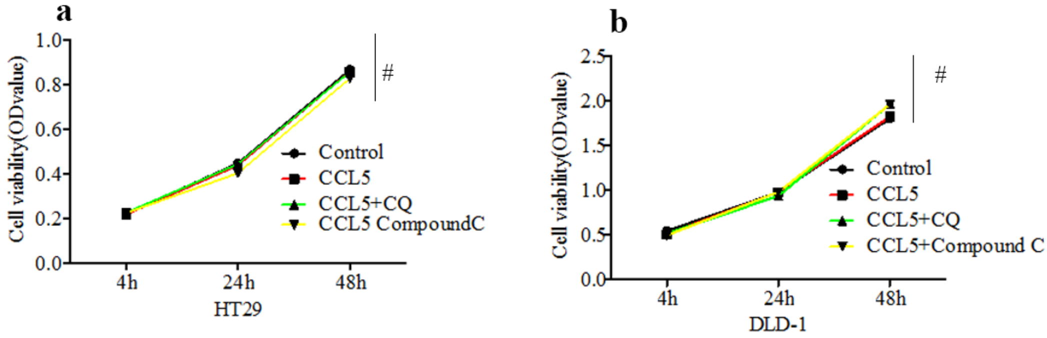

Supplement 7 Blocked autophagy has no effect on HT29 and DLD-1 cells proliferation.. Each data point was the mean±SEM of three independent experiments and presented relative to the control values. （#P>0.05）

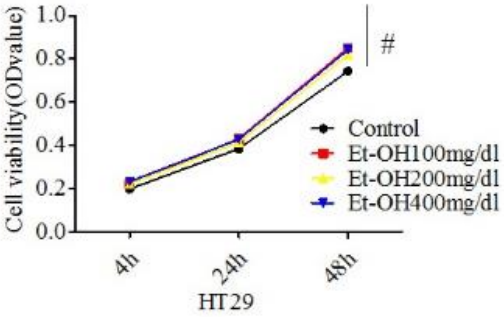

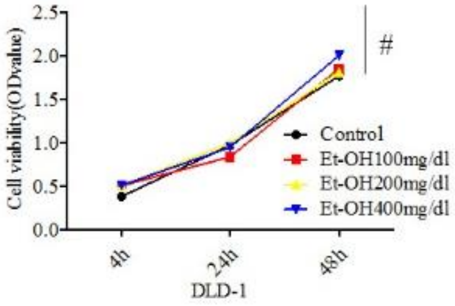

Supplement 8 Et-OH has no effect on CRC cells proliferation（#P>0.05. Each data point was the mean±SEM of three independent experiments and presented relative to the control value.（#P>0.05 n=3）
